# Significant association of the *CHRNB3-CHRNA6* gene cluster with nicotine dependence in the Chinese Han population

**DOI:** 10.1038/s41598-017-09492-8

**Published:** 2017-08-29

**Authors:** Li Wen, Haijun Han, Qiang Liu, Kunkai Su, Zhongli Yang, Wenyan Cui, Wenji Yuan, Yunlong Ma, Rongli Fan, Jiali Chen, Keran Jiang, Xianzhong Jiang, Thomas J. Payne, Jundong Wang, Ming D. Li

**Affiliations:** 1 State Key Laboratory for Diagnosis and Treatment of Infectious Diseases, The First Affiliated Hospital, Collaborative Innovation Center for Diagnosis and Treatment of Infectious Diseases, Zhejiang University School of Medicine, Hangzhou, China; 2 ACT Center for Tobacco Treatment, Education and Research, Department of Otolaryngology and Communicative Sciences, University of Mississippi Medical Center, Jackson, MS USA; 3 Shanxi Key Laboratory of Environmental Veterinary Medicine, Shanxi Agricultural University, Taigu, China; 4 Research Center for Air Pollution and Health, Zhejiang University, Hangzhou, China; 5 Institute of Neuroimmune Pharmacology, Seton Hall University, South Orange, NJ USA

## Abstract

Although numerous studies have revealed significant associations between variants in the nicotinic acetylcholine receptors (nAChR) subunits and nicotine dependence (ND), only few studies were performed in Chinese subjects. Here, we performed association and interaction analysis for 20 single nucleotide polymorphisms (SNPs) in the *CHRNB3-CHRNA6* gene cluster with ND in a Chinese Han population (N = 5,055). We found nominally significant associations for all tested SNPs with ND measured by the Fagerström Test for Nicotine Dependence score; of these, 11 SNPs remained significant after Bonferroni correction for multiple tests (*p* = 9 × 10^−4^~2 × 10^−3^). Further conditional analysis indicated that no other SNP was significantly associated with ND independent of the most-highly significant SNP, rs6474414. Also, our haplotype-based association analysis indicated that each haplotype block was significantly associated with ND (*p* < 0.01). Further, we provide the first evidence of the genetic interaction of these two genes in affecting ND in this sample with an empirical *p*-value of 0.0015. Finally, our meta-analysis of samples with Asian and European origins for five SNPs in *CHRNB3* showed significant associations with ND, with *p*-values ranging from 6.86 × 10^−14^ for rs13280604 to 6.50 × 10^−8^ for rs4950^.^ This represents the first study showing that *CHRNB3/A6* are highly associated with ND in a large Chinese Han sample.

## Introduction

Tobacco use continues to be a leading cause of preventable morbidity and death worldwide^1^. The World Health Organization predicts that if the current smoking trend continues, the annual number of deaths from smoking-related diseases will double to 10 million in 2020^2, 3^. Because of the effect of legislation against tobacco smoking and public realization of its health consequences, the prevalence of cigarette smoking has declined in the United States and other developed countries^4^. On the other hand, despite heightened public awareness of the negative health consequences, smoking remains prevalent or has even increased in many developing countries^5^, especially in Asian nations such as China. The smoking rate in Chinese men aged 15 or older has been estimated to be 66%^6^, implying that more than 350 million Chinese men smoke tobacco, almost one third of the total smokers in the world^7^.

Cigarette smoking, initiation, nicotine dependence (ND) and cessation^8, 9^, are complex and multifactorial behaviors determined by genetic and environmental factors, as well as their interactions. Evidence from twin studies indicates that the mean heritability of ND is 0.56 in total and 0.59 for male smokers^10^.

Neuronal nicotinic acetylcholine receptors (nAChRs), which are ligand-gated ion channels consisting of five subunits, are the primary targets for nicotine to initiate the brain responses to smoking. Accordingly, it is biologically plausible that the genes encoding these nAChR subunits influence smoking-related behaviors such as initiation, intensity, and ND. During recent years, considerable efforts, especially genome-wide association studies (GWAS), have been conducted to identify genetic factors underlying ND, with several widely accepted successes, including the identification of the importance of AChR subunit gene clusters on chromosomes 15 (*CHRNA5/A3/B4*)^11–16^ and 8 (CHRNB3/A6)^17–20^ and the genes encoding nicotine-metabolizing enzymes on chromosome 19 (CYP2A6/A7)^19, 21–23^. As of October 2015, nine published GWAS and meta-GWASs have yielded 11 genetic loci carrying linked variants (*p* < 5 × 10^−8^) associated with relevant ND phenotypes^16, 19, 21, 24–29^. Among these, three genome-wide significant single nucleotide polymorphisms (SNPs) on chromosome 8p11^19, 27^ (rs13280604, rs6474412, and rs1451240, in perfect linkage disequilibrium; LD) in samples of European and African ancestries have been suggestively associated with the ND status of smokers^30^. Although the dichotomized Fagerström Test for Nicotine Dependence (FTND) appears to have an equivalent relation with rs1451240 across ethnicities, the relation between this SNP and the number of cigarettes smoked per day (CPD) is much weaker in African Americans (AAs) than in European Americans (EAs)^27^. The other two SNPs were both significantly associated with CPD in European samples^19^.

The *CHRNA6* and *CHRNB3* genes are located contiguously in a tail-to-tail configuration on chromosome 8p11. The protein products of the two subunit genes are co-localized in nAChRs in the substantia nigra, ventral tegmental area, striatum, and locus coeruleus^31^. Previous candidate gene-based association studies have found that SNPs in the *CHRNB3-CHRNA6* cluster are significantly associated with the risk of ND and various tobacco-use behaviors^18, 30, 32–38^. For detailed information, please refer to our recent published reviews on this gene family^39–41^.

Although current findings have greatly improved our understanding of the etiology of ND, much is still unknown. In particular, there arises a critical question whether the identified associations are reproducible across different ancestral populations. Considering that nearly all subjects used in GWAS or candidate gene-based association studies were of European origin, it would be interesting to know whether SNPs in *CHRNA6* and *CHRNB3* are also involved in ND in smokers of other ethnicities. Given the demonstrated differences across ethnicities with respect to genetics, physiological processes, and behavior underlying ND^42–45^, it is necessary to conduct this kind of work with samples of other ethnicities, such as Chinese smokers, as we report in this study.

## Materials and Methods

### Subjects

We recruited Han Chinese participants aged 20 years and older from several communities in the cities of Jincheng and Taiyuan, Shanxi Province in 2013. Because few females (~4%) smoke tobacco in China, only males (n = 5055) were included in the study. A structured questionnaire on smoking behavior, demographics, medical history, lifestyle, environment, and dietary characteristics was administered to each subject by trained researchers in local hospitals of the two areas. Individuals with other psychiatric diagnoses, such as major depression or schizophrenia, were excluded. The subjects were biologically unrelated to each other. After a detailed explanation of the project and process of this study, informed written consent was obtained from each participant. The Institutional Review Board of the First Affiliated Hospital of Zhejiang University School had approved the project and all questionnaires used for it, and all experiments were performed in accordance with relevant guidelines and regulations of the institution. Table 1 and Supplementary Figure 1 provides the detailed characteristics of the subjects. We defined non-smokers as persons having smoked fewer than 100 cigarettes during their lifetimes, while smokers were assessed with the FTND^46, 47^, one of the commonly used measures of ND (0–10 scale).

**Table 1:** Demographic and phenotypic characteristics of Jincheng and Taiyuan samples.

Characteristic	Jincheng	Taiyuan	Total
Sample size	2,848	2,207	5,055
Smoking rate (%)	51.4	52.2	51.7
FTND in smokers only (SD)	5.14 (1.42)	5.83 (1.30)	5.45 (1.41)
Age, years (SD)	36.4 (9.6)	41.8 (10.6)	38.8 (10.4)
BMI (SD)	24.4 (3.1)	24.0 (3.2)	24.2 (3.1)
Years of working (SD)	11.1 (9.2)	14.3 (12.1)	12.5 (10.7)
No. of smoking family member (SD)	1.7 (0.7)	1.8 (0.9)	1.8 (0.8)

### SNP selection and genotyping

**Figure 1:**
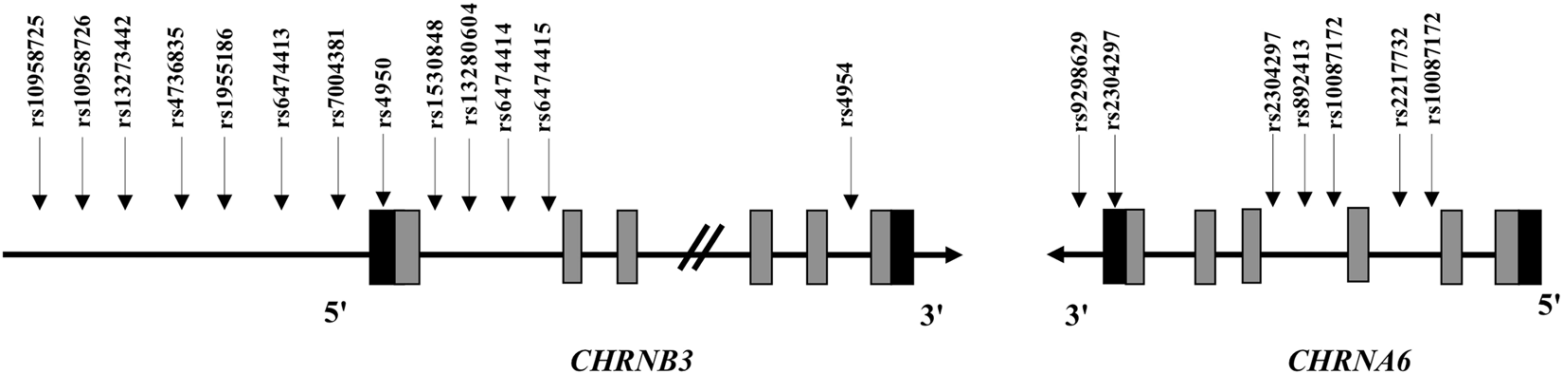
Schematic diagram of the human *CHRNB3*-*CHRNA6* cluster for selective SNPs (not drawn to scale). Horizontal black arrows indicate the direction of transcription of each gene. Gray and black rectangles indicate exons separated by intronic regions and untranslated regions, respectively. The 20 SNPs that were genotyped are shown by vertical arrows, with their rsID and gene locations indicated.

Twenty SNPs in the *CHRNB3-CHRNA6* cluster (*CHRNB3*: rs10958725, rs10958726, rs13273442, rs4736835, rs1955186, rs6474413, rs7004381, rs4950, rs1530848, rs13280604, rs6474414, rs6474415, rs4954; *CHRNA6*: rs9298629, rs2304297, rs892413, rs10087172, rs2196128, rs2217732, rs1072003) were selected using the SNP Browser software from Applied Biosystems (http://www.appliedbiosystems.com) by searching dbSNP (http://www.ncbi.nlm.nih.gov/SNP/) and other published papers^17, 18, 34, 35, 37, 48–50^. The SNPs were chosen to span the two genes and to have moderate minor allele frequencies (MAF ≥ 5% in CHB (Chinese Han in Beijing) based on the NCBI SNP database) and to assay reliably (Supplementary Table 1; Fig. 1).

Genomic DNA was extracted from the peripheral blood of each participant using the Qiagen DNA purification kit according to the manufacturer’s instructions. The concentration of DNA in each sample was determined by the optical density (OD) at 260 nm, and the quality was evaluated by the OD 260/280 ratio. SNP genotyping was performed by using the *Taq*Man OpenArray Genotyping Platform (Applied Biosystems, Inc.). It requires a pair of primers common to both wild-type and mutant sequences, as well as two minor groove binder probes, one being a probe for the normal sequence, labeled with the VIC fluorophore, and the other being a probe for the mutant sequence, labeled with the FAM fluorophore. For the samples with an A_260/280_ ratio between 1.7 and 2.0 and a DNA concentration of 50 ng/μl, about 2 μl (ca. 100 ng) of each genomic sample was placed in a well of a 384-well plate, together with 2 μl of OpenArray Master Mix, using a distribution pattern according to the manufacturer’s instructions. The sealed plate was then centrifuged briefly at 1000 rpm to remove any bubbles. The samples, tips, and the OpenArray plate (composed of 96 sub-arrays, each with 64 nano-wells) were placed in the AccuFill system, and the plates were customized to provide an even distribution. A total volume of 33 μl was dispensed into each nano-well. After the plates had been stabilized with the immersion fluid in a glass case, they were sealed with a photosensitive gum for 2 min under ultraviolet light in the sealing station. The amplification reaction was performed in the Dual Flat Block GeneAmp PCR System 9700 thermal cycler, a process that lasted approximately 4 h. The plates were then placed in an OpenArray NT Cycler for detection of the emitted fluorescence. After capture of the signal, the results were imported into *Taq*Man Genotyper software for SNP calling.

Quality control (QC) tests were performed on each DNA sample, with the goal of excluding samples with sex or chromosomal anomalies, a low call rate, or first- or second- degree relatedness. With this QC step, we removed 334 samples with a call rate of <95%. The genotyping rate for all tested SNPs was >95%, and the *p* value for the Hardy-Weinberg equilibrium was set to >0.05.

### Statistical analysis

#### Individual SNP-based association analysis

For individual SNP-based association analysis, we used the PLINK program (v. 1.07)^51^ to perform linear regression for FTND score under an additive genetic model with age, body mass index (BMI), miner (whether the subject had worked as a miner or not), work years, and number of family members who smoke as covariates. We also performed association analysis by excluding “number of family members who smoke” as a covariate from the analysis, which yielded almost the same association results (data not shown). Statistically significant results (*p* < 0.05) for individual SNPs were adjusted for multiple testing using Bonferroni correction.

#### Haplotype-based association analysis

Pair-wise LD and haplotype blocks were assessed by Haploview (v. 4.2)^52, 53^, and their associations with the FTND score were analyzed using Haplo Stats (v. 1.6.8) through computing score statistics with the same covariates and genetic models used in the individual SNP-based association analysis^54^. The significance of each haplotype was adjusted by the number of major haplotypes (frequency > 5%) included in the analysis, a strategy used to find out if detected associations represent the same signal or not, as we did previously^15, 37, 55^.

#### Gene-gene interaction analysis

To further determine the genetic contribution of the *CHRNB3-CHRNA6* cluster to ND, we performed a gene–gene interaction analysis for the total sample as we did previously for nAChR genes *CHRNA5/A3/B4* ^14, 15^, *CHRNA4*, and *CHRNB*2^56^. We employed the GMDR-GPU program, which was developed by our group^57^, for the SNP-by-SNP interaction analysis of *CHRNB3* and *CHRNA6* with exhaustive searches by two- to five-way SNP combinations. The GMDR-GPU is an improved version of the original GMDR program^58^ that can run much faster because of the integration of more efficient computational algorithms and capacities. Similar to the association analysis described above, GMDR-GPU calculated a score statistic for each subject based on a generalized linear model under an assumption of normal distribution of the FTND scores. We used the following three criteria to determine the best statistical gene–gene interaction model: the cross-validation consistency (CVC), the prediction accuracy, and the significant *p* value, which was determined by 10^7^ permutation tests for the selected SNP combinations.

#### Meta-analysis of individual SNP-based association with ND

We searched PubMed and Web of Science for all studies eligible for this meta-analysis according to the PRISMA guidelines^59^, a strategy similar to what we used previously^60, 61^. The key words used in the search were “*CHRNB3*” or “*CHRNA6*” or “cholinergic nicotinic receptor gene” combined with “nicotine dependence” or “ND.” The reports containing the chosen words were checked individually for references that could have been missed by the above-mentioned search protocol. The inclusion criteria for meta-analysis were as follows. First, the study presented sufficient information for sample size, effect size of the tested allele and *p* values. Also, each study had to be independent, with no duplication or overlap. To combine the published data with the association results from this study, we meta-analyzed each SNP individually under the random-effects model by METAL^62^. Heterogeneity among different ethnic samples was assessed with*I* ^2^ in METAL, which measures the degree of inconsistency among the results from different samples^63^.

## Results

### Individual SNP-based association analysis

As shown in Table 2, the MAF and the β of the SNPs tested for association with ND were almost the same for the Jincheng and Taiyuan samples. Because of the lack of heterogeneity between the samples from the two sites on the basis of principal component analysis (PCA), we calculated the linear regression of each SNP using the total sample with an additional covariate “site.” Of the 20 SNPs analyzed, 10 in *CHRNB3* and 1 in *CHRNA6* showed significant association with FTND after Bonferroni correction for multiple testing by SNP number, with the *p* value for rs6474414 being the smallest one. Further, we performed conditional analyses on rs6474414, which revealed no other SNPs that are significantly associated with ND.

**Table 2:** Associations of SNPs in the *CHRNB3-CHRNA6* cluster with FTND score.

Gene	SNP	Position	Location	Minor allele	Jincheng			Taiyuan			Total		
					MAF	Beta	*p* value	MAF	Beta	*p* value	MAF	Beta	*p* value
*CHRNB3*	rs10958725	42524584	5′ near gene	T	0.258	−0.18	0.0613	0.262	−0.12	0.3248	0.260	−0.15	0.0519
	rs10958726	42535909	5′ near gene	G	0.225	−0.26	0.0117	0.225	−0.27	0.0336	0.225	−0.26	**0.0015**
	rs13273442	42544017	5′ near gene	A	0.224	−0.25	0.0133	0.225	−0.28	0.0250	0.225	−0.26	**0.0013**
	rs4736835	42547033	5′ near gene	T	0.226	−0.25	0.0131	0.224	−0.28	0.0260	0.225	−0.26	**0.0012**
	rs1955186	42549491	5′ near gene	C	0.226	−0.25	0.0139	0.226	−0.28	0.0281	0.226	−0.26	**0.0015**
	rs6474413	42551064	5′ near gene	C	0.226	−0.25	0.0153	0.226	−0.28	0.0247	0.226	−0.26	**0.0014**
	rs7004381	42551161	5′ near gene	A	0.227	−0.25	0.0169	0.228	−0.27	0.0309	0.227	−0.25	**0.0020**
	rs4950	42552633	5′ UTR	C	0.226	−0.25	0.0140	0.225	−0.28	0.0272	0.226	−0.26	**0.0014**
	rs1530848	42552908	Intron	C	0.232	−0.26	0.0119	0.231	−0.27	0.0321	0.231	−0.26	**0.0015**
	rs13280604	42559586	Intron	G	0.227	−0.25	0.0138	0.225	−0.28	0.0248	0.226	−0.26	**0.0012**
	rs6474414	42560336	Intron	A	0.226	−0.27	0.0266	0.226	−0.28	0.0090	0.226	−0.27	**0.0009**
	rs6474415	42562938	Intron	G	0.224	−0.24	0.0210	0.226	−0.24	0.0556	0.225	−0.23	0.0044
	rs4954	42587796	Intron	G	0.156	−0.34	0.0035	0.157	−0.20	0.1620	0.157	−0.27	0.0033
*CHRNA6*	rs9298629	42606186	3′ near gene	T	0.237	−0.25	0.0125	0.230	−0.18	0.1628	0.234	−0.22	0.0072
	rs2304297	42608199	3′ UTR	C	0.240	−0.27	0.0079	0.229	−0.17	0.1824	0.235	−0.22	0.0059
	rs892413	42614378	Intron	A	0.235	−0.27	0.0115	0.229	−0.26	0.1475	0.232	−0.22	0.0062
	rs10087172	42616868	Intron	C	0.237	−0.28	0.0049	0.230	−0.18	0.1514	0.234	−0.23	0.0033
	rs2196128	42618286	Intron	C	0.238	−0.28	0.0058	0.230	−0.18	0.1617	0.234	−0.23	0.0041
	rs2217732	42618446	Intron	G	0.237	−0.28	0.0061	0.231	−0.18	0.1515	0.234	−0.23	0.0040
	rs1072003	42620001	Intron	G	0.238	−0.29	0.0043	0.233	−0.20	0.1185	0.236	−0.24	**0.0020**

Note: For individual SNP-based association analysis, the β and *p* value were adjusted for age, BMI, whether the person had worked as a miner, years of working, and number of smoking family member. For total sample analysis, “site” was used as the additional covariate. Significant associations are given in bold after Bonferroni correction for the 20 SNPs analyzed in the study (0.05/20 = 0.0025). MAF = minor allele frequency; UTR = untranslated region.

### Haplotype-based association analysis

**Figure 2:**
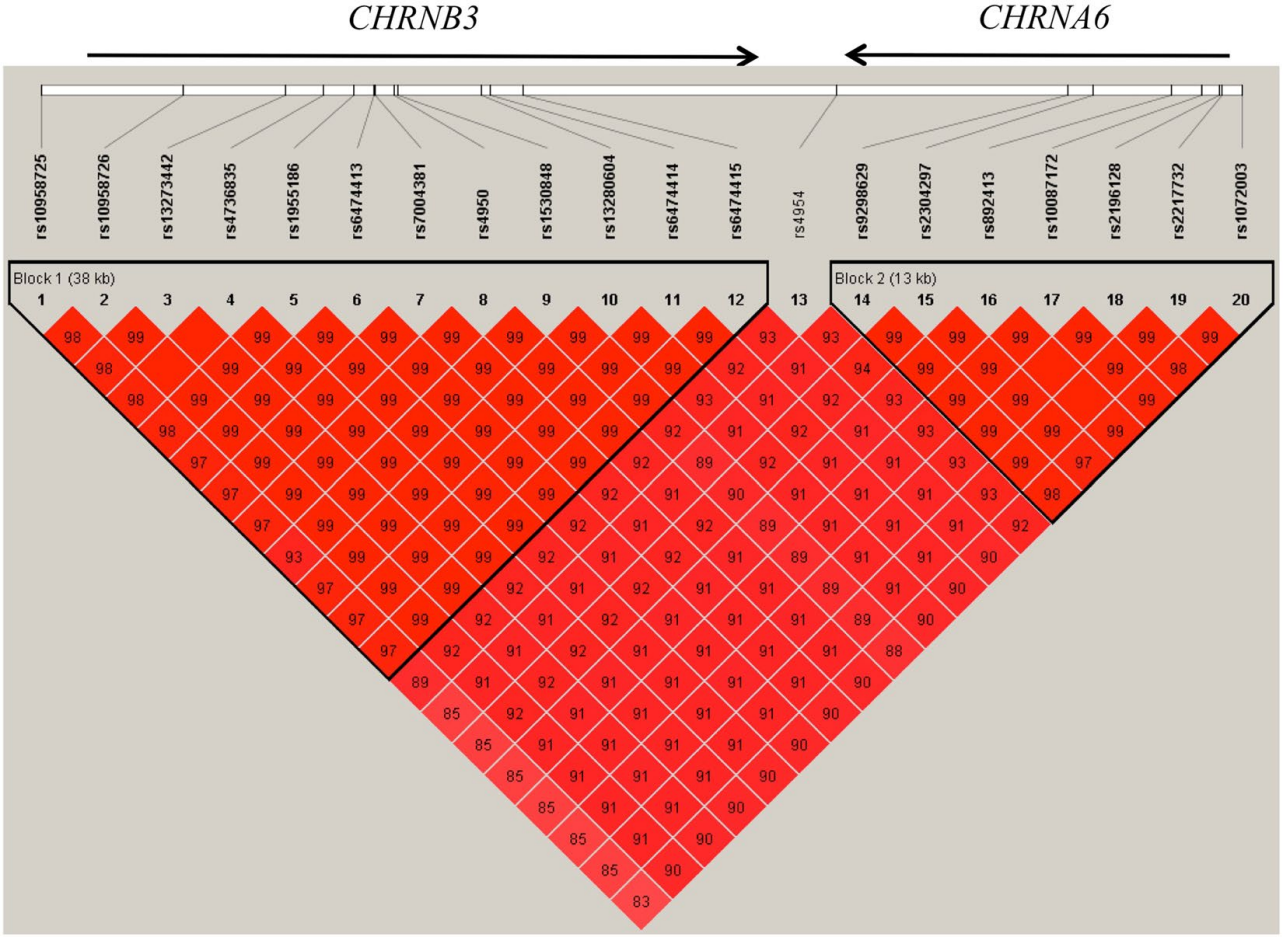
LD structure for *CHRNB3* and *CHRNA6* SNPs in the pooled samples (Jincheng and Taiyuan). Haplotype blocks were defined according to Gabriel *et al*.^52^. The number in each box represents the *r* ^*2*^ value for each SNP pair as calculated by Haplotype (v. 4.2). The arrow on top of the figure represents the gene transcription direction from 5′ to 3′.

Haplotype-based association analysis was performed for all SNPs in the *CHRNB3-CHRNA6* cluster, regardless of the individual SNP-based association results. Similar to the results of the individual SNP-based association studies, we revealed no heterogeneity for the haplotype patterns detected at the two sites. According to the haplotype block criteria defined by Gabriel *et al*., only one block (D′ > 0.97) of *CHRNB3* as well as *CHRNA6* was identified with the pooled samples, and each site-specific sample (Fig. 2) appeared only once for the same block. Because the major haplotypes detected in each block were the same for the two sites, we present the association results for pooled samples.

In *CHRNB3*, one haplotype of T-G-A-T-G-C-A-G-G-G-A-G, formed by SNPs rs10958725, rs10958726, rs13273442, rs4736835, rs1955186, rs6474413, rs7004381, rs4950, rs1530848, rs13280604, rs6474414, and rs6474415, with a frequency of 21.8%, was significantly associated with FTND (Hap Score = −2.8; *p* = 0.005) (Table 3). In *CHRNA6*, we found two significant haplotypes, T-C-A-C-C-G-G and G-G-C-T-T-A-C, with a frequency of 22.9% and 76.2%, respectively, formed by the SNPs rs9298629, rs2304297, rs892413, rs10087172, rs2196128, rs2217732, and rs1072003. These two associations remained significant after Bonferroni correction for multiple testing (Hap Scores = −2.8 and 2.6; *p* = 0.006 and 0.009, respectively) (Table 3).

**Table 3:** Association results of major haplotypes (frequency ≥ 0.05) in the *CHRNB3-CHRNA6* cluster with FTND score in the total sample.

Gene	SNP combination	Haplotype	Freq.	Haplotype Score	Haplotype *p* value
*CHRNB3*	rs10958725-rs10958726-rs13273442-rs4736835-rs1955186-rs6474413-rs7004381-rs4950-rs1530848-rs13280604-rs6474414-rs6474415	T-G-A-T-G-C-A-G-G-G-A-G	0.218	−2.8	**0.005**
		G-T-G-C-C-T-G-A-T-A-C-A	0.731	1.8	0.07
*CHRNA6*	rs9298629-rs2304297-rs892413-rs10087172-rs2196128-rs2217732-rs1072003	T-C-A-C-C-G-G	0.229	−2.8	**0.006**
		G-G-C-T-T-A-C	0.762	2.6	**0.009**

Note: Analysis of haplotype score and *p* value were adjusted for age, BMI, miner, years of working, number of smoking family members, and site; Significant associations are given in bold after Bonferroni correction for four major haplotypes (0.05/4 = 0.0125).

### Gene–gene interaction analysis

Based on the fact that *CHRNB3*, which is adjacent to *CHRNA6* (Fig. 1), usually is co-expressed with α6, immunoprecipitation and high-affinity [^125^I] α-conotoxinMII (αCtxMII)-binding studies showed that the α6β3* pentamer was the predominant α6*-nAChRs in the striatum^64, 65^. The accessory subunit, β3, has an essential role in α6*-nAChRs biogenesis and function^66, 67^. Thus, there might be epistatic effects in ND between *CHRNB3* and *CHRNA6*. Consistent with this hypothesis, we found the best interaction model of 5 SNPs in two genes (rs10958725 and rs1955186 in *CHRNB3*; rs10087172, rs2196128 and rs1072003 in *CHRNA6*), showing a CVC of 10/10, had a prediction accuracy of 52.71% and an empirical *p* value from 10^7^ permutations of 0.0015 (Table 4).

**Table 4:** Best interaction model for *CHRNB3-CHRNA6* cluster with FTND score in Chinese Han samples.

No. of Loci	Best Model	CVC	Prediction Accuracy	*p* value
5	*CHRNB3*: rs10958725, rs1955186 *CHRNA6*: rs10087172, rs2196128, rs1072003	10	52.71%	0.0015

Note: CVC = cross validation consistency. Empirical *p* value was calculated by 10^7^ permutations; in GMDR-GPU analysis, age, BMI, work as a miner, years of working, and number of family member smokers, and site were used as covariates.

### Meta-analysis of individual SNP-based association with ND

Table 5 presents the association analysis results of each SNP among all samples, which include minor allele, β value, SE, and *p*. Because of the high heterogeneity between the ancestries of African and European or Asian (i.e., minor alleles of all five SNPs in African population were opposite to those in the European and Asian populations), we excluded AAs from our meta-analysis. The four samples included in this study were from: (1) the Chinese sample used in the current study; (2) the Korea Association Resource (KARE) study^68^; (3) European Americans (EAs) from MSTCC^20^, SAGE^69^, and CGEMS^70^; and (4) other EAs from the ENGAGE, TAG, and Ox-GSK consortia^19^. Because no FTND score was available for a few samples, the ND phenotype was measured by CPD^15^. Meta-analysis was performed for the five SNPs by combining the results from the included studies. Of the meta-analyzed SNPs, rs13280604 in CHRNB3 (*p* = 6.86 × 10^−14^) showed the strongest association with ND.

**Table 5:** Meta-analysis results for association of five SNPs in *CHRNB3* with ND.

SNP	Minor Allele	Chinese Sample (N = 5,055)			Korean Sample (N = 1,970)		European-American Sample 1 (N = 5,616)		European-American Sample 2 (N = 76,957)		Meta-analysis (N = 89,598)
		β (SE)	*p* value		β (SE)	*p* value	β (SE)	*p* value	β (SE)	*p* value	Combined *p-*value
rs10958725	T	−0.15 (0.08)	0.0519		−0.18 (0.07)	0.0137	−0.22 (0.05)	3.55E-05	−0.29 (0.05)	8.50E-08	8.89E-12
rs10958726	G	−0.26 (0.08)	0.0015		−0.18 (0.07)	0.0097	−0.24 (0.05)	1.39E-05	−0.30 (0.05)	1.70E-08	1.01E-13
rs4950	G	−0.26 (0.08)	0.0014		−0.18 (0.07)	0.0104	−0.24 (0.05)	1.07E-05	−0.21 (0.06)	6.00E-04	6.50E-8
rs13280604	G	−0.26 (0.08)	0.0012		−0.18 (0.07)	0.0109	−0.23 (0.05)	1.41E-05	−0.31 (0.05)	1.30E-08	6.86E-14
rs6474415	G	−0.23 (0.08)	0.0044		−0.18 (0.07)	0.0113	−0.23 (0.05)	1.97E-05	−0.30 (0.05)	2.90E-08	4.17E-13

SE = Standard Error.

## Discussion

In the current study, we examined genetic associations and epistatic effects in *CHRNB3-CHRNA6* linked to ND in the Chinese Han population. Individual SNP-based association analyses revealed that 11 SNPs in this region showed a significant association with the FTND score after Bonferroni correction for multiple testing. Of these polymorphisms, rs6474414 in *CHRNB3* showed the strongest association (*p* = 0.0009) in the pooled sample. Then we found one haplotype in *CHRNB3* (*p* = 0.005) and two haplotypes in *CHRNA6* (*p* = 0.006 and 0.009) that were associated significantly with FTND in the total sample. Furthermore, considering the fact that the protein products of these nAChR subunit genes must join in order to form functional nAChRs, we also performed gene–gene interaction analysis on all SNPs in this genomic region and found that the best interactive model involved 5 SNPs of the *CHRNB3-CHRNA6* cluster. Finally, by conducting meta-analysis of published samples with Asian and European ancestry, we obtained confirmation of the previous findings and further promoted the genetic signals linking *CHRNB3* with ND.

There are several highlights of this study. First, although both GWAS^19, 30^ and candidate gene-based association studies^18, 32, 37^ have implicated variants in the *CHRNB3-CHRNA6* cluster in the development of ND, almost all subjects used in these studies were of European ancestry. Meanwhile, the reported largest GWAS of smoking with the African American samples did not identify this locus to be significant^26^. Given the known ethnic differences in physiological processes and behavior underlying ND^42–45, 50^, it is of great interest to determine whether this gene cluster is associated with ND in smokers of other populations, especially Asians. To attack this issue, we conducted the present study designed to investigate the association of candidate genes with ND in a Chinese Han population. To the best of our knowledge, this is the first genetic study of ND considering FTND score as a quantitative trait in a large Chinese sample.

Second, we provided additional support for a role of the *CHRNB3-CHRNA6* cluster in mediating ND by meta-analyzing samples of smokers with different ethnicities, based on the hypothesis that the biological mechanisms underlying a disease of interest are shared by various human populations. There were multiple SNPs significantly associated with a decreased risk of tobacco addiction after Bonferroni correction in *CHRNB3* (*p* value from 9 × 10^−4^ for rs6474414 to 2 × 10^−3^ for rs7004381). Of these, rs10958726 and rs13280604 achieved genome-wide significance (*p* < 5 × 10^−8^) for CPD in the combined analysis of more than 80,000 individuals^19^. Furthermore, these two variants participated in the subjective responses to initial tobacco use^32, 36^, the transition from occasional cigarette smoking to ND^18, 27^, and the number of quit attempts^3^. However, either none or nominal significance was identified regarding the association of these two variants with ND in African-American and Korean samples^17, 71^. By extracting data from other reports, we performed a meta-analysis with samples of Asian and European ancestry. All alleles with negative effects for five SNPs in *CHRNB3* were the same in both ethnic populations. Significant association of ND with *CHRNB3* was present in these ethnic samples, with *p* values ranging from 6.86 × 10^−14^ for rs13280604 to 6.50 × 10^−8^ for rs4950. Four SNPs reached genome-wide significance, providing convincing evidence of a role of *CHRNB3* in ND by analyzing samples of two representative ancestries from throughout the world. Our meta-analysis results indicates that this region is homogeneous across populations, implying any causative variants identified in this gene could be important for almost all smokers, regardless of ancestry, except for AAs.

Third, we not only conducted association analyses of this region with ND at the SNP and haplotype levels, as in most reported studies of the association of the region with smoking, but also performed gene–gene interaction analysis exclusively in this region. As *CHRNB3* and *CHRNA6* lie tail to tail on chromosome 8 and are both components of nAChRs, it is biologically plausible that they function together to influence the etiology of ND. In addition, there are not many loci in *CHRNA6* found to be significantly associated with smoking behavior so far, in contrast to the abundant results of the gene from *in vivo* studies (see below for details). One possible mechanism is the interaction between common variants within the *CHRNB3-CHRNA6* cluster, similar to our previously detected interactions between *CHRNA4* and *CHRNB2* and CHRNA5/A3/B4^14, 56^. In accordance with this hypothesis, we detected two loci in *CHRNB3* and three loci in *CHRNA6* that contributed interactively to FTND in our sample. This is the first evidence of an epistatic effect of these two genes discovered through genetic analysis in any ethnic sample. Statistically, interaction analysis within genes or within multigene regions is necessary to explore the mechanism of the observed association of common variants with complex traits.

Several functional studies by genetic manipulation in rodents have revealed the vital role of the α6 subunit in the establishment of ND despite the absence of evidence at the SNP-based association analysis level^72–74^. For example, α6 wild type (WT) mice self-administered nicotine in a unit dose of 26.3 μg/kg per infusion, whereas their α6 knockout (KO) drug-naive littermates did not. The α6-KO animals did not self-administer nicotine even in an extensive range of lower (8.7–17.5 μg/kg) and higher (35–52.6 μg/kg) doses. Importantly, when the α6 subunit was selectively re-expressed in the VTA of α6^−/−^ mice using a lentiviral vector, the reinforcement property of nicotine was restored^75^. However, most genetic studies tested *CHRNB3-CHRNA6* together in which significant association was more likely attributable to variants in *CHRNB3*. Moreover, the association of *CHRNA6* SNPs typically did not survive correction for multiple testing^32, 35^. In the present study, we demonstrated that rs1072003 in *CHRNA6* was still significantly associated with FTND score after Bonferroni correction (*p* = 0.002). To the contrary, Saccone *et al*.^17, 35^ did not find evidence of an association between this SNP and ND defined as an FTND score ≥ 4 in EA or AA samples, and neither did Zeiger *et al*.^32^ nor Ehringer *et al*.^36^ using subjective responses to tobacco as phenotypes.

By using an *in vitro* culture approach, Ehringer *et al*.^36^ conducted a luciferase reporter assay study for different haplotypes in the *CHRNB3* promoter. Data from these experiments indicated that different alleles in the *CHRNB3* upstream promoter region led to different degrees of RNA expression. Rs4950 dramatically diminished the binding capacity of a specific transcription factor, Oct-1, and greatly reduced the activity of the promoter of *CHRNB3* ^76^. This finding is consistent with the speculation that this SNP might have an effect on the expression of *CHRNB3*, based on prediction by the Transcriptional Element Search System (TESS), a publicly available program^3, 32^. Kamens *et al*.^77^ illustrated that the protective genetic variant at rs6474413, but not rs4950, reduced gene expression and decreased β3 gene expression in mice and reduced nicotine intake. This kind of work increases our understanding of the molecular mechanisms that contribute to the human genetic associations of tobacco use behaviors, making such work highly important.

One or more differences in study design, ascertainment criteria, assessment of ND, demographic variables, or sample size could contribute to differences in the association of SNPs in the nAChR genes with smoking behaviors. For example, in a Han Chinese sample, Wei *et al*.^49^ did not find any significant locus in the *CHRNB3* gene except for rs4954. Although it is difficult for us to explain such a difference, we suspect that small sample size (i.e., 223 cases and 257 controls) used in their study might be one of the main reasons for them to detect any potential significant association. Furthermore, Wei and his associates classified their smokers into low ND (LND; FTND ≤ 3) and high ND (HND; FTND ≥ 6) groups, which differed from the definition of smoking used in our study in which we treated FTND as a quantitative trait. Furthermore, we genotyped more SNPs in the *CHRNB3-CHRNA6* region in comparison of number of SNPs included in their study on this region. More importantly, we performed not only individual SNP- and haplotype-based association analysis but also interaction analysis among the variants within this *CHRNB3*-*CHRNA6* region. Nevertheless, because of the high LD of the SNPs in each gene examined in our study, it remains to be determined which SNP indeed contributes to the significant association signal. As a matter of fact, SNPs involved in the present study are either intronic or outside the translational region of the gene cluster; thus, it is less clear whether one of these SNPs is the functional variant(s) responsible for the observed association. More effort is needed to find causal variant(s) in this region.

To improve detection power and reduce the possibility of population substructure which might influence our association findings, we recruited all our subjects from the same geographic areas and required all enrolled subjects to be Han Chinese and free of other diagnosed psychiatric disorders or using other substances. Using the subjects recruited from the same geographic areas imply that they are more homogeneous and have similar genetic background according to a recent reported study showing that the genetic clustering of Han Chinese is closely related to their geographic location^78^. Because majority of smokers are males in China and many other Asian countries^79^, we only performed our genetic analysis of variants in this cluster with ND in male smokers. We wish that we could not do similar genetic analysis for female smokers as well, which can be considered a potential limitation of this study. Another potential limitation of this study is that we did not genotype as many SNPs as we wish for this region including failed to genotype one of three SNPs with genome-wide significance (i.e., rs1451240). To have better coverage for these samples, more SNPs with a high density are needed for further analysis of this gene cluster in relation to ND. Furthermore, we identified only one block each in the *CHRNB3* as well as in *CHRNA6*, and found that major haplotypes in each of the above-mentioned LD blocks were significantly associated with the FTND score. However, as reported by Saccone *et al*.^17^, there might be at least two distinct loci for ND in *CHRNB3-CHRNA6*. The first one appears to be driven by rs13277254 and other highly correlated SNPs such as rs10958726 and rs1955186. The other one was tagged by rs4952, the only known coding SNP in exon 5 of *CHRNB3*. Because of the extremely low minor allele frequency of rs4952 in Asian populations, we did not detect this signal in our sample. Finally, we failed to find another independent Chinese smoker sample to replicate our detected SNP-SNP interaction results in this paper. Thus, it is important and necessary to replicate our reported gene-by-gene interaction results in future study with independent samples.

In sum, our results indicate that both *CHRNB3* and *CHRNA6* are significantly associated with ND in the Chinese Han population. We derived this conclusion by genetic analysis at both the individual SNP and haplotype level, which made these genes excellent subjects for research on the molecular mechanisms of ND. This study represents a significant extension of earlier reports in which most subjects were of European origin and which promoted the association signals of *CHRNB3* by meta-analysis. Meanwhile, we also detected gene-by-gene interaction between *CHRNB3* and *CHRNA6* statistically, consistent with the hypothesis that these two genes function together in mediating tobacco addiction. A better understanding of the role of these genetic variants will be key for pharmacologic targeting to reduce or possibly eliminate some of the addictive properties of nicotine in susceptible individuals.

## Electronic supplementary material

Supplementary Information 
