# Supplementary material for: Significant association of the CHRNB3-CHRNA6 gene cluster with nicotine dependence in the Chinese Han population
Source: Sci Rep. 2017 Aug 29;7:9745. doi: 10.1038/s41598-017-09492-8 (PMC5575130; doi:10.1038/s41598-017-09492-8)

Significant association of the *CHRNB3-CHRNA6* gene cluster with nicotine dependence in the Chinese Han population

Li Wen, Haijun Han, Qiang Liu, Kunkai Su, Zhongli Yang, Wenyan Cui, Wenji Yuan, Yunlong Ma, Rongli Fan, Jiali Chen, Keran Jiang, Xianzhong Jiang, Thomas J. Payne, Jundong Wang, and Ming D. Li

Supplementary Table 1. Detailed information of selected SNPs in the *CHRNB3-CHRNA6* gene cluster

Gene	dbSNP ID	Assay ID	Chr. Pos.	Location	Alleles (Major/minor)	MAF
	rs10958725	C__11868275_20	8: 42524584	5' near gene	G/T	0.26
	rs10958726	C__11868302_10	8: 42535909	5' near gene	T/G	0.21
	rs13273442	C__11868306_10	8: 42544017	5' near gene	G/A	0.21
	rs4736835	C__26072327_10	8: 42547033	5' near gene	C/T	0.21
	rs1955186	C__11855540_10	8: 42549491	5' near gene	G/C	NA
	rs6474413	C__28976622_10	8: 42551064	5' near gene	T/C	0.21
CHRNB3	rs7004381	C__26072326_10	8: 42551161	5' near gene	G/A	0.21
	rs4950	C___8238995_20	8: 42552633	5'UTR	T/C	0.21
	rs1530848	C___1346774_10	8: 42552908	Intron	A/C	0.20
	rs13280604	C____272767_10	8: 42559586	Intron	A/G	0.21
	rs6474414	C____272765_10	8: 42560336	Intron	C/A	0.21
	rs6474415	C__29352729_10	8: 42562938	Intron	A/G	0.21
	rs4954	C__12110132_10	8: 42587796	Intron	A/G	0.18
	rs9298629	C__29905827_10	8: 42606186	3' near gene	G/T	0.22
	rs2304297	C__15974192_10	8: 42608199	3' UTR	G/C	0.22
	rs892413	C___9179088_10	8: 42614378	Intron	C/A	0.22
CHRNA6	rs10087172	C__29616685_20	8: 42616868	Intron	T/C	0.22
	rs2196128	C__15936015_20	8: 42618286	Intron	T/C	0.23
	rs2217732	C___9179113_10	8: 42618446	Intron	A/G	0.22
	rs1072003	C___8779199_1_	8: 42620001	Intron	C/G	0.22

Note: SNP, single nucleotide polymorphism; Chr. Pos., chromosomal position; MAF, minor allele frequency based on HapMap-HCB; NA, not available.

Supplementary Figure 1. Distribution of FTND scores in the sample

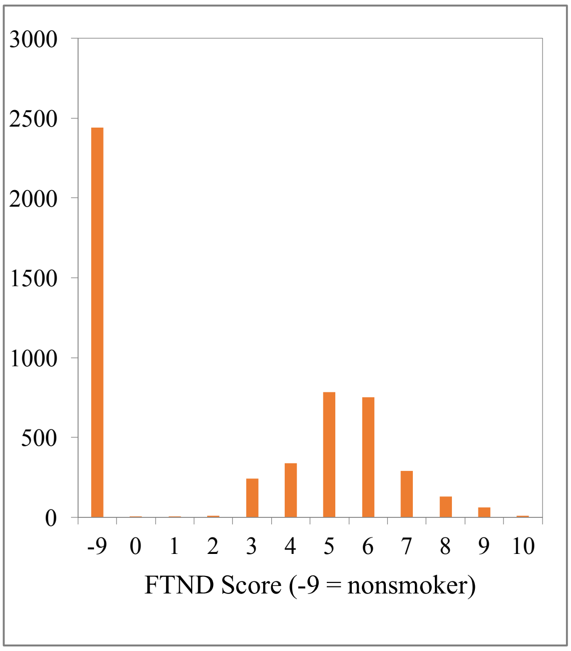

Supplement: Supplementary file 1 — Supplementary Information [file 41598_2017_9492_MOESM1_ESM.pdf]
